# Comparison of Chest-to-Back Skin-to-Skin Contact and Chest-to-Chest Skin-to-Skin Contact on the Risk of Oxygen Desaturation and Change in Heart Rate in Low Birth Weight and/or Premature Babies: A Randomized Controlled Clinical Trial

**DOI:** 10.1155/2021/7196749

**Published:** 2021-12-08

**Authors:** Sisay Gere, Yemane Berhane, Alemayehu Worku

**Affiliations:** ^1^Department of Nursing, College of Health Sciences, Arsi University, Asella, Ethiopia; ^2^Department of Epidemiology and Biostatistics, Addis Continental Institute of Public Health, Addis Ababa, Ethiopia; ^3^Department of Reproductive Health and Population, Addis Continental Institute of Public Health, Addis Ababa, Ethiopia; ^4^Department of Biostatistics, Faculty of Health, Addis Ababa University, Addis Ababa, Ethiopia

## Abstract

Chest-to-chest (CC) skin-to-skin contact (SSC) is a widely used method of SSC to prevent low birth weight (LBW) and/or premature babies with the risk of hypothermia. However, very recently, a study has also shown that the chest-to-back (CB) SSC is also useful for such a purpose. It is also evident that CC SSC enhances the cardiorespiratory performance of LBW and/or premature babies from the risk of cold stress. However, whether babies kept in CB SSC have the risk of clinically relevant decreases of oxygen saturation or critical changes of the baby heart rate comparing the two SSC methods has been studied hardly. Thus, we assessed the risk of oxygen desaturation and changes in babies' heart rate among LBW and/or premature babies kept in CB SSC compared to the standard. In this study, we enrolled 46 LBW and/or premature babies born between 32 and 37 completed weeks of gestation. We used a parallel-group randomized controlled clinical trial. Peripheral arterial blood oxygen saturation (SpO_2_) and heart rate (HR) were measured using an OxiMaxN-600X Pulse Oximeter. We transformed these measurements into stability of the cardiorespiratory system in premature infant (SCRIP) scores. We applied a generalized estimating equation model to analyze the data. No statistically significant difference was observed between babies kept in CB SSC compared to babies kept in CC SSC in either blood oxygen saturation or heart rate (*P* > 0.05). Thus, the CB SSC can be used as one possible way to care for LBW and preterm babies in the kangaroo mother care. We suggest more studies before scaling up the approach in routine care.

## 1. Background

Skin-to-skin contact (SSC) is the major component of the kangaroo mother care (KMC) in neonatal care [1, 2]. The benefits that SSC has for infants range from improving survival to better breastfeeding practices [3–5] in low-income settings [6]. The commonly used SSC method is chest-to-chest (CC) [2, 7]. However, we have shown that a chest-to-back (CB) SSC has a comparable effect in protecting infants from the risk of hypothermia [8]. The CB method was argued to be more feasible to enhance the mother's adherence as it interferes less with the daily chores of the mother in cultures where babies are usually carried at the back of the mother [8].

Low birth weight (defined by the World Health Organization as weight at birth of <2500 grams) and/or premature babies are highly vulnerable to the risk of hypothermia and other untoward effects of cold stress [9–11]. The cold stress depletes their oxygen saturation capacity causing bradycardia, apnea, and cyanosis [12–15]. These conditions can be fatal [16–18]; hypoxia (oxygen saturation below 80%) [19–21] coupled with mild/moderate hypothermia increases the risk of death [22, 23].

The CC SSC stabilizes the heart rate, respiratory rate, and oxygen saturation in LBW and/or premature babies [24–26]. However, whether similar effects can be obtained using chest-to-back SSC was not assessed before. Thus, this study was conducted with the aim of assessing the oxygen desaturation and changes in heart rate in LBW and/or premature infants comparing CB SSC to CC SSC.

## 2. Materials and Methods

The study was part of a large research project, which was registered in ClinicalTrials.gov with an identification code of NCT04346498. The study was conducted in Ethiopia [27]. The details of the methods are presented in our previous paper [8].

### 2.1. Study Setting

The setting was Arsi University Asella Teaching and Referral Hospital Neonatal Intensive Care Unit (NICU) KMC room. It was located in the Oromia regional state of Ethiopia. The total beds available in the neonatal ICU rooms were 26, and the total beds available in the KMC room were four [28]. The setting promotes SSC for all hemodynamically stable babies. It also promotes SSC to families of infants receiving palliative care within the NICU. Continuous Positive Airway Pressure (CPAP) service is also available in the setting.

### 2.2. Participants

We enrolled babies born between 32 and 37 completed weeks of gestation and/or birth weight of ≥1000 grams up to less than 2500 grams.

### 2.3. Ethical Concerns

The study was approved by the Arsi University Ethical Review Committee with reference number A/CHS/RC/15/16. And it was publicly registered in ClinicalTrials.gov with the identification code NCT04346498 [27]. A designated research coordinator recruited eligible families into the study after describing the study thoroughly (risk/benefits, voluntary participation, and procedures). Families were also given adequate time (at least a day) to reflect on the information given. Any question was answered, and written consent was received freely and voluntarily. Those who attested their voluntariness and passed the screening criteria were involved in the trial. No identifiable information is published.

### 2.4. Trial Design

A parallel-group randomized controlled clinical trial was applied.

### 2.5. Randomization

Babies were randomly assigned into arm 1 and arm 2 based on “born on odd-days” and “born on even-days” of a month [8].

### 2.6. Intervention

**Figure 1:**
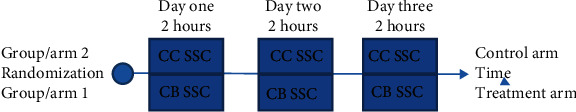
Schematic representation of the intervention.

Our babies' assigned to arm 1 received the CB-SSC (the alternative to the standard of care), and those babies assigned to arm 2 received the CC SSC (the standard of care in kangaroo mother care). In arm 1, a naked chest newborn was positioned upright on the naked back of the mother between her scapulae in a direct SSC. In arm 2, a naked chest newborn was positioned upright in a direct SSC with the naked chest of the mother between her breasts. In either arm, participants were kept for two hours a day for 3 consecutive days while their oxygen and heart rate were monitored continuously (Figure 1).

Infants of both arms were clothed with the same type of diaper, warm hat, and socks. Babies and mothers were also wrapped in the same kind of clothes in both arms. Furthermore, room temperature, chronological age, and weight of babies were measured as described in the previous paper [8].

### 2.7. Outcome Measures

The peripheral arterial blood oxygen saturation (SpO_2_) and heart rate (HR) of babies were the outcome variables. We used the OxiMaxN-600X Pulse Oximeter to measure the study outcomes. SpO_2_ was expressed in percent, and HR was expressed in beats per minute (bpm). The device we used was a CE (FDA) approved device (Re: K123581). Its brand name is Nellcor. It is produced by the CovidienllC, 6135 Gunbarrel Avenue, Boulder, CO 80301 [29].

Our time frame for measuring the outcomes was 2 hours every day for 3 consecutive days. For each outcome, therefore, each study subject was monitored continuously for 120 minutes a day for 3 consecutive days. This gives us 360 person minutes per variable per subject. Multiplying 360 by the number of subjects produced 8280 person minutes per variable in either arm.

Then, we transformed the measurements (the original continuous scale) into stability of the cardiorespiratory system in premature infant (SCRIP) scores during analysis. This was to account for the subnormal ranges in the risk determination [24, 30, 31]. As shown in Table 1, the SCRIP score is an ordinal scale. For instance, perfect stability in SPO_2_ means oxygen saturation of 90% and above (it has 2 points).

### 2.8. Sample Size

Using the G∗power [32], we estimated the sample size for this study with the following assumptions: a possible correlation of 0.105 among repeated measures, an effect size of 0.25, alpha error 0.05, power 0.8, and number of group 2. Thus, the minimum required sample size was 23 babies per group.

### 2.9. Statistical Methods

We used SPSS version 21.0 (IBM Corp., Armonk, NY, and USA) software to analyze our data. We used a generalized estimating equation (GEE) [33–35] model. Specifically, we used a multivariable ordinal logistic GEE model for oxygen saturation and a multivariable binary logistic GEE model for the heart rate. The reason for using binary logistic GEE for the heart rate data was attributed to the lack of cases in the “severe instability” category of the outcome variable. Thus, we merged the “severe instability” level and the “minor instability” level into one. Therefore, we have had two outcome levels for the heart rate (perfect stability and minor instability); thus, a binary logit multivariable GEE link was used. For both outcome variables, the covariance structure we chose for the repeated measure was robust and the working correlation selected was autoregressive type one (AR1). Both outcome variables were also adjusted for sex, birth weight, gestational age, chronological age, room temperature, and breastfeeding/suckling status of the babies, to mention.

## 3. Result

**Figure 2:**
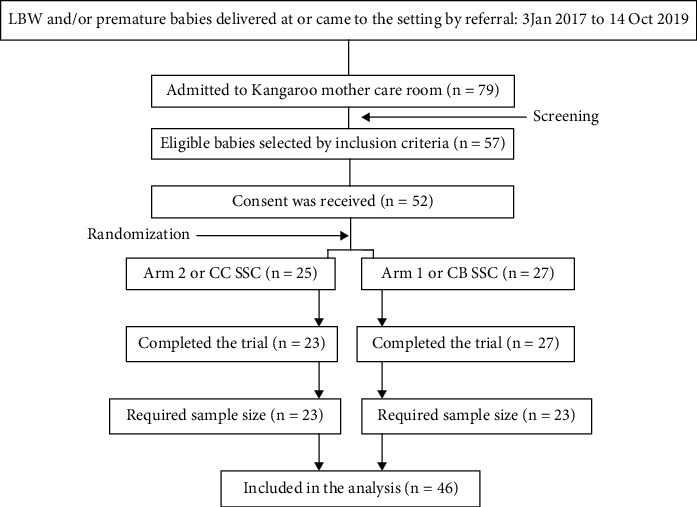
Flow of participants.

Figure 2 shows the flow of the participants in the study; a total of forty-six mother-to-baby pairs (23 from each group) were included in the analysis of this study.

The basic characteristics of the study participants are displayed in Table 2. The two arms were comparable in terms of birth weight, weight during the trial, and mode of delivery of the babies (*P* > 0.05). However, the groups differ by the sex of babies, gestational age, and suckling status of the babies (*P* < 0.05).

The overall incidence of SPO_2_ desaturation below 90 percent was larger in the CC group (39%) than in the CB group (29.5%) (Table 3). Similarly, the overall occurrence of changes in heart rate was larger in the CC group (1.6%) than in the CB group (0.4%) (Table 3).

**Figure 3:**
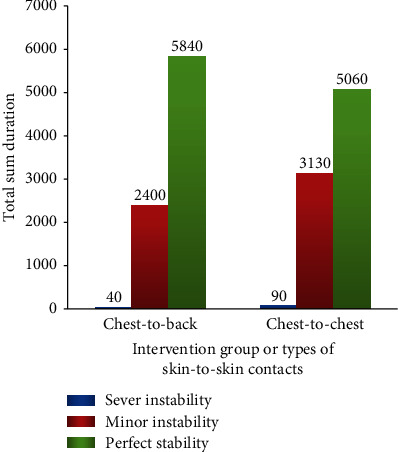
Occurrence of severe instability, minor instability, and stability of oxygen by intervention groups.

Severe instability in HR (i.e., HR < 80 or >180) was not observed in either of the groups. Hence, all changes observed in HR (Table 3) were minor instabilities (i.e., deceleration of HR to 80-99 bpm). However, we observed a severe oxygen desaturation (i.e., fall of SpO_2_ < 80%) in both arms of the trial, which was larger in the CC group (1.1%) than in the CB group (0.5%) (Figure 3).

However, the differences were not statistically significant when adjusted for potential confounders (95% CI AOR 0.919-2.94; *P* value 0.07) (Table 4).

Similarly, after controlling for confounders, no statistically significant difference was observed between the arms on deceleration of HR (95% CI AOR 0.001-1.63; *P* value 0.08) (Table 5).

### 3.1. Trial-Associated Adverse Effects and Complications

In this study, we did not come across trial-associated adverse effects, major complications, and/or deaths.

## 4. Discussion

This study showed that neither the risk of clinically relevant decrease of oxygen saturation nor the risk of changes in infants' heart rates had significant differences between CB and CC skin-to-skin contacts (*P* > 0.05).

Our findings are in accordance with the biology of oxygen consumption of human tissues or cells. Babies can use energy and oxygen to generate warmth when they get cold. For instance, if the body temperature of the baby drops just by one degree from the ideal (97.7°F (36.5°C)), then oxygen use increases by 10% [10, 11, 17]. In cold stress, therefore, oxygen consumption increases as the baby tries to stay warm, which results in tissue hypoxia and consecutive bradycardia [36, 37]. When babies have a normal body temperature or if they are kept in a stable warm condition, their oxygen saturation remains in a clinically acceptable range. Skin-to-skin contact provides that condition; the front body skin and the back body skin of an adult human being have identical thermal comfort [38]. We have also shown that the CB SSC and CC SSC have similar warming effects [8]. Studies also show that CC SSC stabilizes the oxygen saturation and the heart rate of babies in a clinically acceptable range [3, 25, 39]. In this study, we also found no statistically significant difference between CB SSC and CC SSC baby care practices in both oxygen saturation level and the heart rate of babies. Hence, LBW and/or premature infants can be protected from oxygen desaturation and changes in heart rate by CB SSC, if that is a more culturally acceptable mode in the local context.

In general, a stable oxygen saturation and heart rate are essential for the aerobic metabolism, growth, development, and survival of babies [40, 41]. However, because of birth disadvantages [42], LBW and/or premature infants exhibit potentially adverse SPO_2_ desaturation and bradycardia than their counterparts [18, 20, 21]. Thus, finding such a similarity between the two SSC techniques is encouraging as the CB SSC has less interference with the daily chores of the mother and has more acceptances in some cultures, which is the case in Ethiopia [43]. Such culturally acceptable SSC technique is likely to increase adherence for a prolonged kangaroo mother care and can help to reduce the unacceptably high mortality [44–46] in low-income countries [47].

In this study, we did not observe any clinically relevant hypoxemia. The observed hypoxia was most likely physiological and related to body temperature regulation [48, 49]. However, the result should be interpreted cautiously and deserves closer subgroup analysis with a larger sample size in future work. One should also note that although this study has some contributions to the cardiorespiratory parameters of babies cared for in CB SSC, the study did not include all cardiorespiratory parameters required. Thus, future works should also consider this gap and include all cardiorespiratory parameters including apnea.

## 5. Conclusion

In our patients, the use of CB SSC instead of CC SSC was not associated with increased risk of oxygen desaturation and changes in heart rate. Thus, the CB SSC may be used to protect LBW and/or premature infants from the risk of cold stress such as hypoxia. We recommend further studies before scale-up.

## Figures and Tables

**Table 1:** Transformation of the outcomes from continuous scale to ordinal or SCRIP score.

SPO_2_	HR
Perfect stability or regular means ≥90% (has 2 points)	Perfect stability or regular means ≥100 bpm to ≤180 bpm (has 2 points)
Minor instability means any falls to 80%-90% (has 1 point)	Minor instability means deceleration to 80 bpm-99 bpm (has 1 point)
Severe instability means any falls below 80% (has 0 points)	Severe instability means <80 bpm or >180 bpm (has 0 points)

**Table 2:** The CB and CC groups by general character of the participants.

Variables		CB (*n* = 23)	CC (*n* = 23)	*X* ^2^/*t*	Sig
Mode of delivery	Normal/vaginal	17 (74%)	18 (78.3%)	0.583	0.45
	Cesarean section	6 (26%)	5 (21.7%)		
Birth weight in gram (mean ± SD)		1477 ± 0.23	1479 ± 0.16	-0.178	0.86
Gestational age in weeks (mean ± SD)		33.7 ± 1.32	33.6 ± 1.15	2.47	0.01
Gender	Female	13 (56.5%)	9 (39%)	50	0.03
	Male	10 (43.5%)	14 (61%)		
Age category in days	0-7 days	6 (26.1%)	5 (21.7%)	204	0.01
	8-15 days	7 (30.5%)	5 (21.7%)		
	16-21 days	5 (21.7%)	8 (34.9%)		
	≥22	5 (21.7%)	5 (21.7%)		
Weight in gram (mean ± SD)		1483 ± 0.23	1467 ± 0.12	1.79	0.07
Breastfeeding status	Not suckling at all	3 (13%)	4 (17.4%)	150	0.02
	Not suckling effectively	11 (47.8%)	13 (56.5%)		
	Suckling effectively	9 (39.2%)	6 (26.1%)		

**Table 3:** The total sum duration of SPO_2_ desaturation below 90% and the deceleration of HR below 100 bpm in the CB and CC groups.

Outcomes		CB SSC Frequency (%)	CC SSC Frequency (%)
SpO_2_ in %	Regular or > 90 (perfect stability)	5840 (70.53%)	5060 (61.11%)
	Desaturation below 90	2440 (29.47%)	3220 (38.89%)
HR in bpm	Regular or ≥100 to ≤180 (perfect stability)	8250 (99.64%)	8150(98.43%)
	Deceleration below 100	30 (0.36%)	130 (1.57%)

**Table 4:** Comparison of desaturation of SPO_2_ in the two arms: CB SSC vs. CC SSC.

Model terms		AOR	95% CI for AOR		Sig
			Lower	Upper	
Threshold0 (severe instability)		0.019	0.004	2.01	0.26
Threshold1 (minor instability)		0.309	0.017	2.17	0.81
SSC	CB	1.64	0.919	2.94	0.07
	CC	Ref			
Sex	Female	0.973	0.534	1.77	0.93
	Male	Ref			
The breastfeeding status	Not suckling effectively	1.08	0.874	1.33	0.48
	Suckling effectively	Ref			
Gestational age		0.962	0.796	1.162	0.68
Birth weight		1.648	0.390	6.967	0.49
Age of the baby		1.018	0.982	1.054	0.33
Room temperature		1.12	1.056	1.19	0.00
Skin-to-skin contact at a time (*t*)		0.602	0.391	0.926	0.02

**Table 5:** Comparison of changes in heart rate: CB SSC vs. CC SSC.

Model terms		AOR	95% CI for AOR		Sig
			Lower	Upper	
Intercept		3.36	0.12	7.70	0.10
SSC	CB	0.03	0.001	1.63	0.08
	CC	Ref
Sex	Female	2.18	0.34	4.23	0.15
	Male	Ref			
Gestational age		0.71	0.31	1.59	0.40
Age of baby		1.08	0.93	1.26	0.28
Birth weight		0.86	0.25	2.55	0.24
Room temperature		0.24	0.09	0.66	0.005

## Data Availability

Upon a reasonable request, we can offer access to the datasets used and/or analyzed. This can be available from the corresponding author.
